# First Steps to Anatomy

**Published:** 1846-04

**Authors:** 


Art. I.—*First Steps to Anatomy.* By James L. Drummond, m.d., Professor of Anatomy and Physiology in the Royal Belfast Institution.—*London,* 1845. 12mo, pp. 201. With Twelve Plates.

The *idea* of this little volume is excellent; its aim being "to prepare the young medical student, by some initiatory broad views of the general component parts of the animal frame, that he may thereby be better enabled to enter on the innumerable and minute details of physiology and descriptive anatomy." In many respects the execution is correspondingly good. The style is very simple, as befits the object; and the general arrangement of the subjects well fitted to lead on the student by progressive steps towards the higher and more difficult departments of anatomical and physiological science. The chief fault we have to find in it lies in this: that it is, in many respects, decidedly behind the present state of science. We see no more reason why this fault should be excused in an elementary work than in one intended for the more advanced student; since it appears to us to be above all things essential that those who are entering upon any department of study should be made to *learn* as little as possible what they may afterwards be called upon to unlearn. We must justify our statement by a few extracts:

> "While the principle of life thus resists the decomposing operations of chemistry, we must recollect that it is only the injurious part of chemical action which is resisted, and so far is life inimical to this action, that it is only when life itself would be endangered that it is opposed and forbidden; for many chemical processes are carried on in the living body. Among these I may mention respiration and digestion; but these are all for good, and those chemical affinities only, which would tend to injure or destroy the organization, are forbidden to exert their power." (p. 20.)

We have on various former occasions endeavoured to expose the fallacy of this doctrine, (a very *prevalent* one we admit it to be, but not the more true or philosophical,) that the vital power in any way controls chemical change; and the evidence is continually becoming more definite and irresistible, that all these peculiar powers termed vital (and we may instance those of the muscular and nervous systems as most unequivocally falling under this category) are dependent for their manifestation upon chemical changes, which are destructive to the tissues exhibiting them; just as the manifestation of electric power in a voltaic battery is dependent upon the chemical changes taking place between the metals and fluids in its cells. We shall not now dwell further on this topic, since we could scarcely do so without entering upon the question *in extenso,* which we have already discussed as fully as we deem suitable to the nature of the subject.

In the second chapter we have our old friend the cellular substance; which we are informed, as of old, constitutes "the basis of every solid part of an animal." Surely Dr. Drummond must be aware that anatomists, in this country at least, have now universally agreed to change the old designation of the tissue for one which more satisfactorily expresses its character—namely, *areolar* tissue; and that the real cellular tissues of the body, whose extent and actions have been ascertained by the aid of the microscope, perform functions of far greater importance. The functions of the true areolar tissue we conceive to be purely mechanical; it ties together the different elements of other tissues, in such a manner as to permit them to have a certain degree of movement upon one another; but of its being "the matrix or mould in and by which every structure is imbedded, pervaded, or enclosed," we believe that there is an utter deficiency of proof. In cartilage, for instance, not a trace of it is to be found. In bone it only lines the medullary canals and cancelli, serving to fix the vessels in their places; in teeth it does not extend beyond the pulp cavity, where it answers the same purpose; and there are many other textures of which the same may be said. Nor is it correct to say that it forms the chief bulk of the tendons and ligaments, since, as Messrs. Todd and Bowman have shown, the areolar tissue contains an admixture of two elements—the *white* and the *yellow* fibrous structures; of which the former alone is found in the tendons and non-elastic ligaments, whilst the latter is found only in the elastic ligaments. Again, in the succeeding chapter on fat, not a word is said of the important office to which this tissue is subservient in the living body—that of serving as a reservoir of combustible material, by which our heat is kept up, when from any cause there is a suspension of the supply afforded by the food. The experiments of Chossat prove that, if this reservoir be entirely exhausted, the animal is dependent, from hour to hour, upon the combustible matter ingested and absorbed; and thus, if it were not for such a provision, the loss of a single dinner, to say nothing of a longer abstinence, would be fatal. Some little notice of the more precise views, on these and other subjects, which have of late contributed to raise physiology to a much higher rank among the sciences, would greatly improve the value of Dr. Drummond's treatise, in this and many other departments; and such we hope to see introduced in another edition.

In a concluding chapter, entitled 'Thoughts on Anatomical Education,' Dr. Drummond urges what has often been suggested by ourselves as likely to prove advantageous—that the entire first year's course should be of a general preparatory nature, embracing no one subject in detail, but of a nature to lay a broad and solid foundation for the more special inquiries to be subsequently pursued.

